# Febrile neutropenia: significance of elaborated screening for respiratory viruses, and the comparison of different sampling methods, in neutropenic patients with hematological malignancies

**DOI:** 10.1186/1743-422X-10-212

**Published:** 2013-06-27

**Authors:** Rogier R Jansen, Bart J Biemond, Janke Schinkel, Sylvie M Koekkoek, Richard Molenkamp, Menno D de Jong, Caroline E Visser

**Affiliations:** 1 Department of Medical Microbiology, Academical Medical Centre, University of Amsterdam, Amsterdam, the Netherlands; 2 Department of Hematology, Academical Medical Centre, University of Amsterdam, Amsterdam, the Netherlands; 3 Academical Medical Centre, Room L1-245, Meibergdreef 9, 1105 AZ, Amsterdam, the Netherlands

**Keywords:** Respiratory, Virus, Quantitative, PCR, Neutropenia

## Abstract

**Background:**

During febrile neutropenia in only 30 to 60 percent an infectious agent is identified. This diagnostic gap could hypothetically be reduced with the broad implementation of molecular detection techniques like PCR, which has revolutionized the detection of infectious diseases during the last two decades.

**Findings:**

We performed a longitudinal prospective study (N = 81) of neutropenic patients to assess the role of respiratory viruses in neutropenic fever and to determine the clinical relevance of blind screening for these viruses. Respiratory viruses were recovered in 14% of the patients prior to neutropenia. In 13% of neutropenic patients without fever and in 19% of those with fever, a respiratory virus was detected. Comparing different sample types; nasal swabs performed significantly better (16/117 = 43%), than throat swabs (6/106 = 6%). Throat gurgles did not show significant differences from the latter sample types.

**Conclusions:**

Blind diagnostic screening for respiratory viruses before or during neutropenia is not useful. Nasal swabs are sensitive and practical option for screening on respiratory viruses.

## Introduction

Fever during neutropenia is a frequent problem in patients with hematological malignancies and is the most common cause of non-malignant morbidity and mortality in this patient group [1]. An infectious etiology is identified in only 30 to 60 percent of the febrile neutropenic episodes [2-4]. Despite this diagnostic gap, most studies and guidelines merely presume bacterial and fungal microorganisms as the cause of neutropenic fever, but do not extensively look at possible viral etiologies [2-5]. Since respiratory viruses are known to be the most common cause of fever in the general population and the clinical presentation of a viral infection is often not specific in neutropenic patients, diagnostic screening for respiratory viruses seems rational in patients presenting with neutropenic fever. In children, there is growing evidence that neutropenic fever is frequently associated with respiratory viral infections [6,7]. Moreover, in stem cell transplantation (SCT) patients, viruses are recognized to frequently cause post-transplant fever [8-11].

Over the last decade molecular techniques, such as polymerase chain reaction (PCR), have revolutionized the diagnosis of viral infections. PCR has enabled rapid and highly sensitive detection of a large number of respiratory viruses, including those that cannot, or are difficult, to culture, such as human Bocavirus (hBoV) and human metapneumovirus (HMPV) [12-14]. In patients with hematological malignancies, PCR has shown to be far more sensitive than viral culture [11,15], but these studies have not looked into viral prevalence during neutropenic fever.

This study was performed to look at the role of respiratory viruses in neutropenic fever in adults with a hematological malignancy and assess the usefulness of screening for respiratory viruses before the onset of neutropenia.

## Methods

This paper describes a prospective study performed at the Academic Medical Centre (AMC), which acts as tertiary referral centre in the Netherlands. Data was obtained by a longitudinal cohort study at the adult hematology ward of the AMC and was performed from November 2008 until April 2010. Patients were eligible for inclusion when chemotherapy-induced significant neutropenia (i.e. > 7 days) was expected. A patient could be included for every successive neutropenic episode, assuming that the latter episode was followed by full recovery of the peripheral blood neutrophil count and the patient had been discharged from the hospital in between episodes. Three types of respiratory specimens (a nasal swab, pharyngeal swab and throat gurgle) were collected at 3 different time points: upon admission (i.e. around the onset of chemotherapy), during deep neutropenia (10–14 days after onset of chemotherapy) and, if present, at the start of neutropenic fever. The 3 different time points were labeled as phase A, phase B and phase C respectively. Fever was defined as a single temperature of >38.5°C or a sustained temperature of >38°C for more than one hour. Each patient’s symptoms and signs during admission were registered on a case record form. All materials were analyzed by quantitative real time multiplex PCR covering the following viruses; influenzavirus A (InfA) and B (InfB), enterovirus (EV), adenovirus (AdV), respiratory syncytial virus (RSV), rhinovirus (RV) and human-metapneumovirus (hMPV), parainfluenzavirus 1–4, human-parechovirus (hPeV), human-coronavirus (hCoV: HKU1, NL63, 229E and OC43) and human-bocavirus (hBoV) [16,17]. This was done at the end of the study; hence treating physicians were not aware of the study results during patient treatment. The local ethics committee approved the study and all participating patients provided written informed consent before entering the study.

## Results

### Patients characteristics and symptomatology

This study prospectively included 66 patients who developed a total of 81 episodes of neutropenia and 42 episodes of neutropenic fever during the study. In ten of these 81 episodes, sampling at the onset of chemotherapy could not be performed and merely samples during neutropenia were collected. Of the included 81 neutropenic patients (phase A) 42 developed neutropenic fever (phase C). Most of them (n = 35) developed fever before day 10–14 after onset of chemotherapy, and therefore passed over sampling at phase B. Patient characteristics are shown in Table 1. Acute myeloid leukemia was the most frequent diagnosis, followed by multiple myeloma and relapsed diffuse large cell non-Hodgkin lymphoma. Most patients were treated with intensive chemotherapy or myeloablative chemotherapy followed by autologous stem cell rescue or by allogeneic stem cell transplantation. A limited number of patients who were treated with reduced intensity conditioning followed by allogeneic stem cell transplantation were included.

**Table 1:** Patient characteristics

	**Group A**	**Group B**	**Group C**
Number of neutropenic episodes	**N = 81**	**N = 46**	**N = 42**
No individual patients	66	41	38
Age (IQR^5^)	57 (49–64)	56 (38–64)	59 (50–65)
Sex female (%)	34 (42%)	14 (30%)	24 (57%)
Hematological disorder:			
AML (%)	36 (44%)	17 (37%)	23 (55%)
CML (%)	3 (4%)	1 (2%)	2 (5%)
ALL (%)	8 (10%)	8 (17%)	1 (2%)
CLL (%)	3 (4%)	3 (7%)	0
HL (%)	2 (2%)	0	2 (5%)
NHL (%)	11 (14%)	7 (15%)	4 (10%)
MM (%)	14 (17%)	9 (18%)	7 (17%)
Other (%)	4 (5%)	2 (4%)	3 (7%)
Treatment type:			
Chemotherapy (%)	-	29 (63%)	29 (69%)
AutoSCT (%)	-	11 (24%)	12 (29%)
AlloSCT r.i. (%)	-	2 (4%)	1 (2%)
AlloSCT m.a. (%)	-	4 (9%)	1 (2%)
T cell reducing medicine (%)	-	3 (6%)	3 (7%)
Explicit Mucositis (%)	-	24 (52%)	25 (60%)
Duration of neutropenia at sampling (IQR^5^)	-	6 (5–8)	8 (3–12)
COPD (%)	1 (1%)	1 (2%)	0
Smoking (%)	7 (9%)	4 (9%)	3 (7%)
URTS^a^ (%)	15/71^6^ (21%)	6 (13%)	9 (24%)
LRTS^b^	0	0	1 (2%)
Pulmonary aberration’s on imagery (%)	-	2 (4%)	10/32^c^ (31%)
Probable Aspergillosis (%)	-	0	3 (7%)
Positive Blood cultures (%)	-	-	12^d^ (29%)

Group A; Baseline population at onset of chemotherapy, Group B; Patients with deep neutropenia, without fever, Group C; Patients with deep neutropenia and fever . *IQR* Inter quartile range, *AML* Acute myeloid leukemia, *CML* Chronic myeloid leukemia, *ALL* Acute lymphoblastic leukemia, *CLL* Chronic lymphoblastic leukemia, *HL* Hodgkin lymphoma, *NHL* Non Hodgkin lymphoma. *AutoSCT* Autologous stem cell transplantation, *AlloSCT r.t.* Allogeneic stem cell transplantation reduced intensity, regimen, *AlloSCT m.a.* Allogeneic stem cell transplantation myeloablative regimen. *RTS* respiratory tract symptoms.

^a^*URTS* Upper respiratory tract symptoms (i.e. rhinitis, sneezing, ear/nose congestions).

^b^*LRTS* Lower respiratory tract symptoms (i.e. dyspnea, or coughing in combination with auscultation of the lung suggesting bronchitis/pneumonia, or coughing in combination with abnormalities on lung imagery, suspected for infection.

^c^ Respiratory imaging was performed in 32 of the patients.

^d^ Eleven of 12 bloodcultures contained bacteria normally found on the skin (mainly coagulase-negative staphylococcae); in most of the cases this was interpreted by the clinician as a possible central venous catheter infection.

Respiratory tract signs or symptoms were defined as rhinorrhoea, coughing, sneezing, sore throat (except in the neutropenic phase), dyspnea, and crackles or wheezes at auscultation. Sore throat during neutropenia was not considered an infectious respiratory symptom since this was usually associated with chemotherapy-induced mucositis. Respiratory tract complaints were present in 21% (15/71) of patients upon admission (phase A), in 13% (6/41) of patients during neutropenia (phase B), and in 24% (10/42) of patients during neutropenic fever (phase C). A probable clinical diagnosis for neutropenic fever was established by the treating physician in 23/42 (55%) of febrile patients. The 3 most prevalent clinical diagnoses were central venous catheter related infection 13/42 (31%), neutropenic colitis 3/42 (7%) and pneumonia 3 (7%). In contrast to clinical diagnosis, 28% (9/32) of the patients with neutropenic fever had abnormalities on imaging possibly consistent with lower respiratory tract infections.

### Microbiological findings

Diagnostic results are shown in Table 2. Before the start of chemotherapy (phase A), viral nucleic acids were detected in 14% (10/71) of patients. In 13% (6/46) of neutropenic patients without fever (group B) and in 19% (8/42) of those with fever (phase C) a respiratory virus was detected by PCR. The distribution of viral species found in the different groups is described in Table 2. RV was the most frequently detected virus followed by hBoV and hCoV. InfA & B and PIV 2 & 4 were not present in any of the tested samples. No substantial differences in the distribution of specific viral species were observed between groups. Viral positivity did not show any seasonal pattern for any of the groups. Patients with respiratory symptoms were not associated with a higher prevalence of respiratory viruses compared to asymptomatic patients. (resp. 2/10 [20%] vs. 6/32 [19%]).

**Table 2:** PCR results in relation to patient characteristics

	**Phase A**	**Phase B**	**Phase C**
	**N = 71**	**N = 46**	**N = 42**
Viral positivity (percentage)	10/71 (14%)	6 (13%)	8 (19%)
Viral positivity in persons:			
with URTS	1/15 (7%)	1/6 (16%)	1/9 (11%)
with LRTS	0	0	1/1 (100%)
without RTS	9/56 (16%)	5/40 (13%)	6/32 (19%)
Viral positivity in persons:			
with suspected imagery^a^	-	-	1/10^9^ (10%)
without suspected imagery^a^	-	-	4/22^9^ (18%)
Nasal swab Positive (%)	6/57 (11%)	5/35 (14%)	5/25 (20%)
Pharyngeal swab Positive (%)	2/51 (4%)	2/33 (6%)	2/22 (9%)
Throat gurgle positive (%)	5/71 (7%)	4/42 (10%)	5/35(14%)
Frequencies of detect viral species:			
RV	4 (6%)	1 (4%)	3 (7%)
RSV	1 (1%)	1(4%)	0
hMPV	0	0	1 (2%)
hBoV	2 (3%)	0	2 (4%)
PeV	1	0	0
AdV	1	0	1 (2%)
hCoV	1	2 (8%)	1 (2%)
PIV2	0	2	0
PIV3	0	1 (4%)	0

Phase A; Baseline population at onset of chemotherapy, notice that ten patients could not be sampled before the start of chemotherapy (N = 71), Phase B; Patients with deep neutropenia, without fever, Phase C; Prospective patients with deep neutropenia and fever. *URTS* upper respiratory tract symptoms, *LRTS* lower respiratory tract symptoms, *RTS* respiratory tract symptoms, *RV* rhinovirus, *RSV* respiratory syncytial virus, *hMPV* human metapneumovirus, *HBoV* human bocavirus, *PeV* parechovirus, *AdV* adenovirus, *hCoV* corona virus, *PIV 2&3* para-influenza virus 1,2,3,4. *CP-value* crossing point value, *IQR* inter quartile range.

^a^ x-ray or CT-scan of the thorax.

^b^ The following viuses were not detected: Inf A/B; influenza viruses A and B, EV; enterovirus, para-influenza virus 1&4.

Looking at different sample types, nasal swabs had a positivity rate of 14% (16/117), compared to 6% (6/106) for throat swabs and 9% (14/148) for throat gurgles. The difference between nasal swabs and throat swabs was statistically significant (p < 0.05 chi square test). When multiple samples were taken, more than one sample was positive in 49% of the paired samples.

Longitudinal analyses of prospective samples showed that of the 10 viruses found before the start of chemotherapy only 2 were still present during neutropenia (one in a patient with, and another in a patient without, neutropenic fever). In both cases it concerned RV.

## Discussion

Clinical management of neutropenic fever remains a challenge during the treatment of hematological malignancies. In these patients, symptoms are often non-specific and an etiology of infection is frequently not established. In the present study, we performed a longitudinal prospective study to address the role of respiratory viruses in causing neutropenic fever and to assess the clinical relevance of diagnostic screening for respiratory viruses during the course of neutropenia.

The data collected in this study does not support a prominent etiological role of respiratory viruses in the development of neutropenic fever. Patients in whom respiratory viruses were detected did not exhibit more respiratory symptoms or signs than patients without respiratory viruses. While this could possibly be explained by altered or non-specific symptomatology in an immunocompromised setting, there also were no differences in the frequency of virus detection in respiratory specimens obtained before and during neutropenia and during episodes of neutropenic fever. Moreover, respiratory viruses that were present in specimens obtained before the onset of neutropenia seldom remained detectable in follow up specimens collected during neutropenia. These observations suggest that elaborate diagnostic screening for respiratory viruses before or during (febrile) neutropenia is not clinically useful. However it should be noted that the virus species detected in our samples were mostly species that are considered to be less pathogenic (e.g. RV, hBoV and hCoV). For example, Inf A was rarely found. The latter fact could partly be explained by an increased awareness and reinforcement of preventive measures during the threat of the newly appeared H1N1 influenza strain in 2009.

Considering the type of sampling, we observed that nasal swabs performed better than throat swabs. To our knowledge, only a few studies have compared nasal swaps to other sampling techniques when testing for respiratory virus by PCR in adults, and none have been performed in adults with hematological malignancies. These studies showed superior performance for nasal swabs compared to pharyngeal swabs [18,19], although, the use of two simultaneous techniques resulted in the highest yield of positive results. Our findings support the latter findings and suggest that the sole use of pharyngeal swabs should be avoided for viral detection in hematological patients. As shown in Table 2, a complete set of nasal swab, pharyngeal swab and throat gurgle was not obtained for all patients, which could possibly introduce a bias when comparing sampling methods. However since every sample was obtained simultaneously with at least one other sample we think this bias is small.

Although routine screening for respiratory is not warranted, in subject with respiratory symptoms viral testing is useful to determine the etiology for the complaints, the start of barrier precautions and the start of adequate treatment in the case of influenza. In these patients testing of lower respiratory tract samples is favorable to upper respiratory samples [20,21]. Our study had the following limitations; our study is performed in a relatively small study group and represents only limited time span. The circulation and the pathogenicity of viruses can differ from year, and therefore the findings in our study cannot be directly extrapolated to future viral respiratory seasons in other clinical settings. A second limitations is that because of the rise of the new pandemic H1N1 in 2009, there was much more awareness for preventive measures around respiratory virus (i.e. hand hygiene on the ward and vaccination among staff and general population) which may have reduced the spread of influenza and other respiratory virus on our hematology ward.

In conclusion, our prospective study does not support the hypothesis that respiratory viruses play an important role in the development of neutropenic fever, and thus screening for respiratory virus before or during neutropenia is not useful.

## Competing interests

The authors declare that they have no competing interests.

## Authors’ contributions

RJ, BB, JS & CV contributed to the development of the research protocol The gathering of informed consent, patients samples and clinical information, was done by RJ & BB. PCR analysis and statistical analysis was perfomend by SK, RM, RJ. The writing of the manuscript was completed by RJ, BB, MD & CV. All authors read and approved the final manuscript.
